# Foot self-care behavior and its predictors in diabetic patients in Indonesia

**DOI:** 10.1186/s13104-020-4903-y

**Published:** 2020-02-01

**Authors:** Yunita Sari, Arif Setyo Upoyo, Atyanti Isworo, Agis Taufik, Annas Sumeru, Dian Anandari, Eman Sutrisna

**Affiliations:** 1 Department of Nursing, Faculty of Health Sciences, Universitas Jenderal Soedirman, Jl.Dr. Soeparno, Kampus Karangwangkal, Purwokerto, Indonesia; 2 Department of Public Health, Faculty of Health Sciences, Universitas Jenderal Soedirman, Purwokerto, Indonesia; 3 Department of Pharmacology, Faculty of Medicine, Universitas Jenderal Soedirman, Purwokerto, Indonesia

**Keywords:** Behavior, Diabetes, Foot self-care, Predictor

## Abstract

**Objectives:**

Previous studies have shown that diabetic foot ulcers are principally associated with improper foot self-care. Since appropriate foot self-care is essential to prevent diabetic foot-ulcers, any factors which can predict foot self-care behavior should be identified. However, until now, foot self-care behavior data and predictors of foot-care behavior in Indonesia remain unclear since such studies on Indonesian diabetic patients is very limited. Therefore, the purpose of this study was to investigate foot self-care behavior and to identify its predictors in Indonesia. The design of this study was cross-sectional. Cluster sampling was used, involving 546 type 2 diabetes mellitus patients registered in 22 primary healthcare centers. The questionnaires used in this study included the Diabetes Distress Scale, Beck Depression Inventory II, Family APGAR, Foot-Care Knowledge and Modified Diabetic Foot Care Behaviors.

**Results:**

Foot self-care behavior and knowledge about foot care were poor. The predictors of foot self-care behavior were age, educational level, diabetes distress, family support, and knowledge. It needs the program to improve foot self-care knowledge and the program to reduce the diabetes distress in Indonesian diabetic patients. In performing of these programs, families should be involved to improve the support toward foot self-care behavior in patients.

## Introduction

Indonesia is one of the top ten countries with diabetes mellitus (DM) patients [1]. It is predicted there will be 14,1 million diabetic patients in Indonesia by 2035 [2]. One of the most feared and serious complications of DM in developing countries are diabetic foot ulcers [3]. Compared with US and worldwide prevalence, which ranges between 1.4% and 5.9%, the prevalence of diabetic foot ulcer in Indonesia is high, since it is 12% in hospitals and 24% in community settings [3–8]. The presence of a diabetic foot ulcer can affect both the physical and psychosocial life domains, resulting in a reduction in the quality of life, and even mortality [9, 10]. Considering the significant impact of diabetic foot ulcers, strategies are needed to prevent their occurrence.

A previous study showed that the occurrence of diabetic foot ulcers was mainly associated with improper foot self-care [11, 12]. Previous studies showed that adequate foot self-care can reduce the total number of hospitalizations, and amputation by 50% [11, 13–16]. Therefore, assessment of predictors of foot self-care are required so that the best intervention can be designed and implemented. However, until now, the study about foot self-care behavior in Indonesian diabetic patients is very limited. Moreover, there is no data about in which skill of foot self-care practice that Indonesian diabetic patients is still lacking. Therefore, the first purpose of this study was to assess the foot self-care behavior in Indonesian diabetic patients.

Previous studies in other countries revealed that the factors which influence foot care behavior include demographic variables like age, educational level, and occupation [17, 18]. Other factors such as peripheral neuropathy [19], diabetes distress [20], family support [21], depression [22], have also been identified as predictors of foot self-care behavior in diabetic patients. Until now, studies about the predictors of foot self-care behavior in Indonesian patients are very limited, and include only small numbers of participants. No studies have been conducted to investigate whether peripheral neuropathy, diabetes distress, family support, and depression, could serve as predictors of foot self-care behavior in diabetic patients in Indonesia. Until now, studies on predictors of foot self-care behavior in Indonesia have only focused on knowledge variable. However, studies which investigated knowledge as a predictor of foot self-care behavior have shown inconsistent findings in Indonesia. Therefore, the second purpose of this study was to investigate whether peripheral neuropathy, diabetes distress, family support, depression and knowledge affect foot self-care in Indonesian diabetic patients.

## Main text

### Methods

This is a descriptive cross-sectional study. The study was conducted between January 2, 2019 and June 29, 2019. Ethical approval was obtained from the Faculty of Medicine, Universitas Jenderal Soedirman, Purwokerto, Indonesia. Prior to collection of data, written informed consent was obtained from each participant. The purpose of the study, methods, risks and benefits of participating were explained to participants. Respondents were free to withdraw from the study at any time.

A cluster sampling method was used in this study, involving 22 clusters (primary health centers) in Banyumas Regency, Central Java, Indonesia. The sample size was calculated with 95% CI, an absolute estimated precision of 3%, a non-response rate of 5% and a design effect of 2. The final calculation of the sample size was 546 participants. The inclusion criteria were as follows: patients who were 18–80 years old with T2DM, with the ability to practice foot self-care and the ability to communicate. The exclusion criteria were as follows: patients with physical disabilities, immobile, with cognitive impairments, or dementia.

The presence of peripheral neuropathy was assessed with the Michigan Neuropathy Screening Instrument (MNSI). MNSI is a validated tool for screening for peripheral neuropathy in the distal extremity. The second part of the MNSI contains an objective physical assessment test. Previous studies have shown that the MNSI has high sensitivity (80%) and specificity (95%), with a PPV of 97% and a NPV of 74% [23]. The total possible score was 8 points. A score ≥ 2 was considered abnormal [23].

Diabetes distress was assessed by means of the Diabetes Distress Scale [24]. This scale consists of 17 items that use the Likert scale. Items associated with distress experienced over the past month were scored from 1 (not a problem) to 6 (a very serious problem). The Diabetes Distress Scale has been validated in Indonesia. The Cronbach’s alpha of the Indonesian version is satisfactory (0.78–0.83) [25].

Depression was assessed by means of Beck Depression Inventory II. This scale has been widely used to assess the severity of subjective depressive symptoms [26]. A total of 21 items were included in this questionnaire. The items were scored from 0 (no) to 3 (severe). Beck Depression Inventory II has been validated in Indonesia, with a Cronbach’s alpha of 0.90 [27].

Family support was assessed based on the Family APGAR [28]. This scale consists of 5 items, scored from 0 (never) to 4 (always). The Cronbach’s alpha of the subscale ranges between 0.63 and 0.83 [28, 29]. The Cronbach’s alpha of the Indonesian version is satisfactory (0.83) [30].

Knowledge about foot self-care was measured by means of a validated foot care knowledge (FCK) questionnaire [19]. Previous studies have shown that the content validity index of this questionnaire is 0.91 [31]. The use of this scale in Indonesia has shown acceptable test–retest reliability, with a range of values of 0.67–1 [31]. The highest possible score was 11.

Foot self-care behavior was measured by means of the Modified Diabetic Foot Care Behaviors (MDFCB) questionnaire [32, 33]. The MDFCB has proven to be reliable, with a Cronbach’s alpha coefficient of 0.81 [32]. The total number of items in MDFCB is 34. The formula used to calculate the score was as follows: standard score = (actual score/the highest possible score) × 100. If the score was less than 60, it was regarded as poor; if the score was between 60 and 80, it was considered as medium; and if the score was more than 80, it was considered good [34].

All data were analysed using SPPS version 23. The demographic variables were assessed using descriptive statistics (means, standard deviation, frequencies). Multivariate regression analysis was conducted to determine the predictors of foot self-care behavior.

### Results

#### Socio demographic characteristics of the study participants

A total of 546 subjects participated in this study. The demographic characteristics are shown in Table 1.

**Table 1:** Demography characteristics of the respondents (N = 546)

Demographic	n	%	Mean ± SD	Range
Age			60.14 ± 9.2	24–88 years
Gender				
Female	417	76.4		
Male	129	23.6		
Marital status				
Married	436	79.9		
Not married	9	1.6		
Divorced/widowed	101	18.5		
Education				
Less than high school graduate	535	97.9		
High school graduate	6	1.09		
Higher than high school graduate	5	0.91		
Occupation				
Farmer	42	7.7		
Non-farmer	137	25.1		
Unemployed/retired	367	67.2		
Income level				
Low income (less than USD$138 a month)	463	84.8		
Middle income (USD$138–USD$177 USD a month)	78	14.3		
High income (higher than USD$177 a month)	5	0.9		
Comorbidities				
Yes	355	65.1		
No	191	34.9		
Diabetes duration				
<1 years	53	9.7		
1–5 years	272	49.8		
6–10 years	130	23.8		
>10 years	91	16.7		
Peripheral neuropathy				
Yes	294	53.9		
No	252	46.1		
Current foot ulcer				
Yes	190	34.8		
No	356	65.2		

*Mn* mean, *SD* standard deviation

#### Foot self-care behavior and knowledge about foot self-care

Concerning foot self-care behavior, the average standard score was 47.4, indicating an overall poor level of foot-care behavior. Responses to questions regarding foot injury treatment and application of foot moisturizer got the lowest standard scores, with average standard scores of 26.3, and 16.3, respectively. Responses with average standard scores below 60 were: examining foot condition (50.7), appropriate footwear (46.3), and foot injury prevention (34.3).

The mean score for foot-care knowledge was 5.33 ± 2.2, out of a maximum possible score of 11, indicating an overall poor level of foot-care knowledge.

#### Predictors of foot self-care behavior

The results of multiple linear regression analysis are shown in Table 2. The predictors of foot self-care behavior were age, educational level, diabetes distress, family support, knowledge about foot self-care. These five variables explained 19.7% of the variance (R = 0.444, R^2^ = 0.197, F = 10.806, p< 0.001).

**Table 2:** Result of multivariate linier regression (N = 546)

Variable	*B*	SE	β	T	*p*	R	R^2^	F
Constant term	7.910	5.969		1.325	0.000	0.444	0.197	10.806
Age	0.124	0.057	0.095	2.182	0.030*			
Gender	− 2.092	1.296	− 0.075	− 1.614	0.107			
Marital status	− 0.118	1.235	− 0.004	− 0.096	0.924			
Education	3.492	1.523	0.101	2.292	0.022*			
Occupation	− 0.003	0.294	0.000	− 0.010	0.992			
Income level	1.794	2.165	0.035	0.829	0.408			
Comorbidities	− 1.195	1.055	− 0.048	− 1.132	0.258			
Diabetes duration	0.885	0.566	0.067	1.563	0.119			
Peripheral neuropathy	− 0.122	0.245	− 0.023	− 0.501	0.617			
Current foot ulcer	3.218	1.706	0.082	1.886	0.060			
Diabetes distress	0.114	0.032	0.164	3.580	0.000**			
Depression	− 0.110	0.088	− 0.056	− 1.250	0.212			
Foot-care knowledge	14.331	2.207	0.276	6.493	0.000**			
Family support	19.948	4.622	0.179	4.315	0.000**			

*p < 0.05, **p < 0.01

### Discussion

This is the first study to reveal that diabetes distress and family support are predictors of foot self-care behavior in Indonesia.

In this study, the average standard score for foot-care behavior was 47.4, which can be categorized as poor. More specifically, the categories which scored poorly were: use of appropriate footwear, examining foot condition, foot moisturizer use, foot injury prevention and treatment categories. The low score in these categories may be due to the fact that most respondents assume that foot examination, foot and skin injury prevention and treatment do not have to be conducted every day. The low score obtained for moisturizer use may be due to the fact that most patients do not know that moisturizer should not be applied between the toes. The low score regarding foot wear might due to the lack of knowledge about use of footwear and the climate of Indonesia. Indonesia is a hot country, and therefore many people prefer to use sandals instead of shoes.

Our study revealed that patients with an educational level higher than high school graduate obtained significantly higher foot care behavior scores than those with low educational backgrounds. Less educated people tend to have less health knowledge, leading to unhealthy behaviors than those with higher educational levels [35, 36]. Considering that most respondents have a low educational background, nurses who design the educational programs should focus on visual and auditory demonstration methods, rather than on written instructions.

Our study found that age was one of the predictor of foot self-care behavior. Our study is not in accordance with a recent study which showed that foot self-care behavior in elderly patients were poor since they have physical difficulties and inadequate knowledge [37]. This difference might be due to that in our study we exclude elderly patients who have any physical difficulties such as patients who have loss of vision and who are immobile. Another reason is that elderly patients in this study might have more knowledge compared with adult patients since they have longer duration of having diabetes.

In this study, most of the patients’ knowledge was categorized as poor. This lack of knowledge can be explained because most diabetic patients in Indonesia do not receive foot care management information. Based on the knowledge, attitude, and behavior theory, knowledge provides the basis for a positive attitude, and a positive attitude powers behavioral change [18, 38]. According to this theory, persons will do something if they believe that it will have a significant value for themselves. The implication of this theory is that nurses should teach persons with diabetes about the complications which can arise if they fail to properly perform foot care. The fear of the consequences of not treating their feet properly will become a motivation for diabetic patients to properly practice foot care.

Another finding from our study was that family support was a predictor of foot self-care behavior. Even though it is generally accepted that the family has an influence in the management of chronically ill patients [36, 39], the role of family support on foot self-care behavior in Indonesia is still unknown. Families should be involved to improve family support toward foot self-care behavior in Indonesian diabetic patients. In Indonesia, family involvement can easily occur since the patient’s extended family usually lives nearby.

In this study, we found that diabetes distress is one predictor of foot self-care practice. However, our study contradicts the conclusions of a recent study by Devarajooh and Chinna [40], which revealed that there was no relationship between diabetes distress and foot self-care. One possible difference between that study and our study is sociocultural differences. Cultural beliefs influence disease perceptions [41].

It is recommended for Indonesian nurses to design new prevention strategies based on the predictor factors found in this study. An educational program is required to improve the knowledge and skill of patients to perform foot self-care. Additional program which is required is a program of emotional/psychological intervention. This program could reduce diabetic distress in diabetic patients. In performing both of these programs, families should be involved to improve family support toward foot self-care behavior in patients.

### Conclusions

This study demonstrated that foot-care behavior in diabetic patients in Indonesia is poor. This study has shown that age, educational level, diabetes distress, family support and knowledge about foot self-care are predictors of foot self-care behavior in Indonesia.

## Limitation of the study

Firstly, the design of this study (cross-sectional) makes it difficult to establish causal relationships. Secondly, this study was conducted in Indonesia, therefore, the results cannot be generalized to other countries. However, our current study has distinctive strengths that are worth noting. It is the first study about foot self-care behavior that involve a large number of diabetic patients in Indonesia, and the first study to reveal that diabetes distress and family support are predictors of foot self-care behavior in Indonesia.

## Data Availability

Datasets of this study are available from the corresponding author up-on request.

## Abbreviations

MNSI: Michigan Neuropathy Screening Instrument
Mn: mean
SD: standard deviation
T2DM: type 2 diabetes melitus
